# Stability of ellagitannins in processing products of selected *Fragaria* fruit during 12 months of storage

**DOI:** 10.1002/fsn3.3172

**Published:** 2022-12-22

**Authors:** Agnieszka Hejduk, Michał Sójka, Robert Klewicki

**Affiliations:** ^1^ Institute of Food Technology and Analysis, Lodz University of Technology Lodz Poland

**Keywords:** ellagitannins, *Fragaria*, *Rosaceae*, stability, strawberry

## Abstract

The aim of the study was to determine the stability of ellagitannins (ETs), ellagic acid, and conjugates (EAC) in unclarified juices and purees produced from two selected berries of the *Rosaceae* family, genus *Fragaria*. A total of 16 ellagitannins have been identified in the products. Stability studies were carried out for 1 year in three temperature variants: −20, 4, and 20°C (±2°C). The quantitative and qualitative determinations of ETs + EAC were performed every 3 months using the HPLC‐DAD and LC–MS techniques. An important element of the research was also the determination of ETs transformations that occur in juices and purees during storage. The stability of ETs + EAC is primarily determined by the temperature and storage time as well as the matrix. After 1 year of storage at −20°C, the sum of ETs and EAC did not decrease in strawberry juice. On the other hand, the decrease was shown at the level of 30 and 20% at temperatures of 4 and 20°C, respectively. In strawberry purees, a decrease in ETs + EAC of 15–28% was recorded during storage in all three temperature variants. After 1 year, in wild strawberry juice, 20–50% of ellagitannin decreased, in puree the decrease in ETs + EAC concentration was 40–54%. With increasing temperature, the percentage of compounds with lower molecular weight increased—from 10 to 14% at temperatures of 4 and 20°C, respectively. At the temperature of −20°C, the proportion of low‐ and high‐molecular‐weight compounds remained stable in each case.

## Graphical abstract

Studies on the stability of ETs in products—unclarified juices and purees have shown that these compounds are susceptible to degradation/oxidation. The stability of ETs depends on the temperature and storage time as well as the matrix.

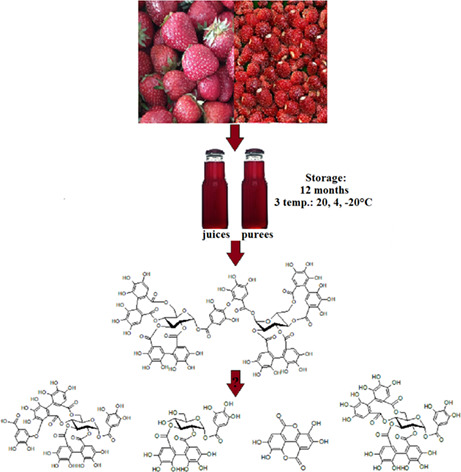

## 1 INTRODUCTION

Berries of the genus *Fragaria*, i.e., strawberries and wild strawberries, contain a high concentration of ellagitannins (ETs)—depending on the variety, from 50 to 300 mg/100 g fresh weight (Gasperotti et al., 2010; Hager et al., 2008; Milczarek et al., 2021). In the processing of selected berries, most of the oligomeric ETs are retained in the pomace (Milczarek et al., 2021). Berries are widely eaten by humans, also in processed form. Due to the low shelf life of berries, they are often frozen and processed into juices, jams, purees, and mousses. Berries, walnuts, and wine are estimated to be the main sources of ETs in the human diet (Clifford & Scalbert, 2000). However, it is not known exactly how many ETs you need to consume to achieve a health‐promoting effect.

Food processing influences the stability of ETs and thus the content of ellagic acid (EA) and its conjugates (Viriot et al., 1993). The presence of free ellagic acid and its derivatives can therefore be an indicator of the degree of processing of a food product. The problem is the inaccurate estimated EA concentrations due to its poor water solubility (Clifford & Scalbert, 2000).

Ellagitannins undergo hydrolysis, degradation, oxidation, and depolymerization reactions (García‐Villalba et al., 2015; Klumpers et al., 1994; Viriot et al., 1994). Hydrolysis of the HHDP (hexahydroxydiphenoyl moiety) releases ellagic acid by cleavage of the ester bond (Clifford & Scalbert, 2000). Methylation and glycosylation of hydroxyl groups lead to the formation of numerous ellagic acid derivatives (Maas et al., 1991). After detaching EA from oligomers, a number of intermediate products are formed, including compounds with a lower molecular weight (Macierzyński et al., 2020; Sójka et al., 2019).

Until now, favorable properties have been attributed to oligomeric ellagitannins (Ko et al., 2015) due to their complex structure. Plant extracts rich in ETs show antimicrobial properties against, e.g., *Escherichia coli*, *Salmonella enteritidis*, *Staphylococcus aureus*, *Bacillus subtilis*, *Klebsiella pneumoniae*, *Alternaria alternata*, *Fusarium oxysporum*, and *Rhizoctonia solani* (Al‐Zoreky, 2009; Ascacio‐Valdés et al., 2013). These compounds have also been shown to inhibit the growth of certain viruses, fungi, and yeasts (Ascacio‐Valdés et al., 2013; Corthout et al., 1991; Klewicka et al., 2016, 2020).

It has been proven that the hydrolysis products of oligomeric ETs, including EA and its conjugates (EAC), have numerous bioactive properties, including antioxidant, anti‐inflammatory, and prebiotic effects (Beekwilder et al., 2005; Kahkonen et al., 2012; Piekarska‐Radzik et al., 2019; Sangiovanni et al., 2013). In in vitro studies, favorable properties are attributed to the degradation products of high‐molecular‐weight ellagitannins. The inhibitory effect of ellagic acid and its further hydrolysis products—urolithin—on prostate cancer cells have been proven (Heber, 2008; Lansky et al., 2005). Moreover, EA is a more accessible compound for microorganisms living in the gastrointestinal tract (Jurgoński et al., 2017).

Determining the degree of ETs and ellagic acid conjugates (EAC) transformation during the storage of fruit products is particularly important for examining their health‐promoting properties. The main aim of the study was to determine the effect of storage time and temperature on the stability of ETs + EAC in unclarified juices and purees of two *Fragaria* fruits. The products were stored at three temperatures: −20, 4, and 20°C (±2°C) for a year. The study also indicates the ratio of high‐ and low‐molecular‐weight ETs during 12 months of storage, as well as changes in the concentration of ETs + EAC characteristic for a given fruit.

## 2 MATERIALS AND METHODS

### 2.1 Fruit

Berries for testing, i.e., strawberry (*Fragaria grandiflora* Ehrh., ‘Sibilla’ cv.) and wild strawberry (*Fragaria vesca* L., ‘Rugia’ cv.), were purchased from the Cajdex company (Lodz, Poland). The fruit was fully ripe. Until processing, the fruit was stored at −20°C in tightly closed polypropylene bags.

### 2.2 Chemicals

Ultrapure water was obtained using the Hydrolab HLP5 system (Straszyn, Poland). The eluents used for HPLC and LC–MS were minimum HPLC grade. Acetonitrile and phosphoric acid were purchased from J.T. Baker (Deventer, The Netherlands). Formic acid was purchased from Sigma‐Aldrich Chemie (Steinhem, Germany) and Chempur (Piekary Slaskie, Poland)—acetone (99.8% purity). Pectinolytic enzyme Pectinex UF was purchased from Novozymes (Bagsvaerd, Denmark). To calculate the amount of ETs, the ellagic acid standard (Extrasynthese, Genay, France) and the ETs standards (minimum 90% purity) produced and purified at the Institute of Food Technology and Analysis of the Lodz University of Technology (Sójka et al., 2016) were used.

### 2.3 Production and storage of unclarified juices and purees

The production of juices and purees was presented in detail in an earlier publication (Milczarek et al., 2021). Fruits in the amount of about 4 kg were thawed at 4°C for 24 h, and then ground using a Zelmer grinding device (Rzeszow, Poland). The ground fruits were incubated for 1 h at 45°C with the pectinolytic enzyme Rohapect Classic (Novozymes, Bagsvaerd, Denmark). The enzyme activity was 1906 U/ml. The enzyme was added at a dose of 0.2 ml/kg. Every 10 min, the pulp was stirred mechanically. After this time, the pulp was divided into two parts. One part was made of unclarified juice using a laboratory hydraulic press (Lodz, Poland). The juice was pressed for 5 min at a pressure of 100 bar. The second part was made into a puree using a steel food mill (Orion, Katowice, Poland). The mesh diameter of the mill was <1 mm. The finished products were subjected to a 2‐min microwave pasteurization using a Daewoo 800 W microwave oven (Pruszkow, Poland). The products were poured hot (85°C) into glass bottles, sealed, and placed in a refrigerator (4°C), a freezer (−20°C), and a temperature‐controlled room (20°C). The preserves were stored in the dark.

### 2.4 Identification of ETs

Two samples (bottles) were taken for analysis every 3 months. Juices and purees were diluted 1:1 (v/v) with an aqueous solution of 60% acetone acidified with 0.1% formic acid. The samples prepared in this way were intended for HPLC‐DAD and LC–MS analyses. Chemical analysis was performed in triplicate.

ETs were identified according to the methodology described by Sójka et al. (2016), using the LC–MS technique. A liquid chromatograph with a DAD detector was used to determine the ETs. This kit was coupled with an Exactive Orbitrap Q mass spectrometer (Thermo Fisher Scientific, Waltham, MA, USA). The separation of the ETs took place on a Luna C18 100 Å, 250 mm × 4.6 mm, 5 mm column (Phenomenex, Torrance, CA, USA). The chromatography column was thermostated at 35°C. The separation of ETs was carried out in a concentration gradient, with phases A (1% aqueous formic acid) and B (80% aqueous acetonitrile). The phases were as follows: 0–6.5 min, 5% of phase B; 6.5–12.5 min, 5—15% of phase B; 12.5–44 min, 15–45% of phase B; 44–45 min, 45–75% of phase B; 45–50 min, 75% of phase B; 50–52 min, 75–5% of phase B; and 52–65.5 min, 5% phase B. The flow rate was set to 1 ml/min and the injection volume was set to 20 μl. The spectra were registered in the negative mode. The detector worked in full MS mode and full scan mode MS/dd‐MS2 (MS with data‐dependent tandem mass spectrometry). In the MS full scan mode, the mass range (*m/z*) was set to 200–2000. The selected precursor ions entered into an HDC collision cell. The capillary and evaporator temperature were set at 400 and 500°C, the impact energy at 20 eV, and ion spray voltage of 4 kV. The resolution value for full MS was 70,000 (mass range 150–2000 *m/z*), and for dd‐MS^2^ it was 17,500 (mass range 200–2000 *m/z*). The auxiliary gas intensities were 72 and 20 units, respectively. Data were collected using Xcalibur 3.0.63 software (Thermo Fisher Scientific Inc.).

### 2.5 Quantification of ETs

The determination of ETs concentration in the processing products of wild strawberries and strawberries was carried out in accordance with the methodology described by Sójka et al. (2016). A Smartline Knauer liquid chromatograph (Berlin, Germany) with a PDA detector (2800) was used for quantification. The separation of the ETs took place on a Gemini C18 100 Å, 250 mm × 4.6 mm, 5 mm column (Phenomenex, Torrance, CA). The flow rate was set at 1.25 ml/min, and the injection volume was 20 ml. The detection of ETs was at a wavelength of 250 nm. The separation took place in a concentration gradient (phase A—0.05% aqueous phosphoric acid solution, and phase B—83% acetonitrile solution in 0.05% phosphoric acid) with the following phases: 0–5 min, 5% phase B; 5–10 min, 5–15% of phase B; 10–35 min, 15–40% of phase B; 35–40 min, 40–73% of phase B; 40–44 min, 73% of phase B; 44–46 min – 73–5% of phase B; and 46–54 min, 5% of phase B. The concentrations of individual ETs were calculated on the basis of standard curves for standards in the concentration ranges: 0.5–225 mg/L for lambertianin C (LOD = 0.4 mg/L and LOQ = 1.3 mg/L, *R* ^2^ = 0.999), 0.5–200 mg/L for sanguiin H‐6 (LOD = 0.3 mg/L and LOQ = 1.0 mg/L, *R* ^2^ = 0.999), 0.5–100 mg/L for agrimoniin (LOD = 0.4 mg/L and LOQ = 1.3 mg/L, *R* ^2^ = 0.998), and 0.5–300 mg/L for ellagic acid (LOD = 0.2 mg/L and LOQ = 0.7 mg/L, *R* ^2^ = 0.998). The concentration of lambertianin C without HHDP unit was calculated from the standard curve of lambertianin C; agrimoniin without HHDP moiety based on the standard curve for agrimoniin; for other ETs based on the sanguiin H‐6 standard curve; and ellagic acid conjugates based on the standard curve of ellagic acid (Milczarek et al., 2021). Isomers of the same compound were added together when peaks occurred several times. Data were collected using ClarityChrom v. 3.0.5.505 software (Berlin, Germany).

### 2.6 Statistics

The obtained results were analyzed using Statistica 12 (StatSoft, Tulsa, CA, USA). Factorial ANOVA and Duncan's post hoc tests were performed. Additionally, it was examined whether there is a correlation (Spearman correlation matrix) between the storage time and temperature and the ETs content.

## 3 RESULTS AND DISCUSSION

### 3.1 ETs in fresh unclarified juices and purees

A total of 16 ellagitannins were identified in juices and purees (Table 1, Table S1 in Supplementary Material section). In all analyzed products, dimer—agrimoniin—, previously indicated as the main ETs in strawberry and wild strawberry fruits, was identified (Milczarek et al., 2021; Vrhovsek et al., 2012). Moreover, in all products, free ellagic acid and an unknown ellagic acid trimer derivative (UEATr) were identified, already indicated as a compound characteristic of wild strawberries (Bubba et al., 2012). The UV spectrum presented in Table 1 does not indicate that this compound contains ellagic acid in its structure. However, only one publication was found indicating this chemical compound as an unknown ellagic acid trimer derivative (Bubba et al., 2012). Bis‐HHDP‐glucose, sanguiin H‐10 isomer, casuarictin, castalagin, and ellagic acid conjugates, which were already indicated as ETs of *Fragaria* fruit, were also identified (Bubba et al., 2012; Milczarek et al., 2021; Vrhovsek et al., 2012).

**TABLE 1:** Identification of ETs

Peak no.	Compound	RT [min]	Λ [nm]	Precursor ion^a^		MS/MS fragment ions *m/z*	Tentative structural assignment	Detected *m/z*	Calculated MM^c^	∆ [ppm]	References
				[M–H]^−^	[M–2H]^2−^						
1	Bis‐HHDP‐glucose/pedunculagin	11.6, 12.8	219	783		481 ([M–H–HHDP]^−^) and **301** (ellagic acid)^b^	C_34_H_24_O_22_	783.0691	783.0687	0.51	Hager et al. (2008)
2	Unknown ellagic acid trimer derivative	13.5	216	947		**901**, 883, 871, 599, and **301** (ellagic acid)		947.0439			Bubba et al. (2012)
3	Sanguiin H‐2 without one gallic acid residue	17.3, 17.4, 17.8, 18.4	208	951		907 ([M–H–CO_2_]^−^), 783 (bis‐HHDP‐glucose), 481 (HHDP‐glucose), and **301** (ellagic acid)	C_41_H_28_O_27_	951.0751	951.0745	0.63	Gasperotti et al. (2010)
4	Agrimoniin without two HHDP moieties	20.0	217	1265		867, 633 (galloyl‐HHDP‐glucose), 613, 574, 461, and **301** (ellagic acid)	C_54_H_42_O_36_	1265.1389	1265.1383		Macierzyński et al. (2020)
5	Sanguiin H‐10 isomer	21.5	213		783	1265 ([M–H–HHDP]^−^), 1103 ([M–H]^−^ – HHDP‐glucose), **633** (galloyl‐HHDP‐glucose), and **301** (ellagic acid)	C_68_H_48_O_44_	783.0688	783.0686	0.26	Gasperotti et al. (2010), Kahkonen et al. (2012)
6	Castalagin type ellagitannin	19.7, 22.2, 22.8	219	933		631, 451, 433, and **301** (ellagic acid)	C_41_H_26_O_26_	933.0656	933.0640	1.72	Bubba et al. (2012)
7	Casuarictin (galloyl‐bis‐HHDP‐glucose)	23.5	213	935		633 ([M−H−HHDP]^−^), 391, and **301** (ellagic acid)	C_41_H_28_O_26_	935.0815	935.0796	2.03	Bubba et al. (2012)
8	Sanguiin H‐6 isomer	23.7	225		934	1567 ([M–H–HHDP]^−^), 1265 ([M–H]^−^–HHDP–HHDP), 1103 (sanguiin H‐2), 633 (galloyl‐HHDP‐glucose), 631 ([M–H–HHDP–HHDP–glucose–galloyl–HHDP]^−^), and **301** (ellagic acid)	C_82_H_54_O_52_	934.0720	934.0718	0.21	Sójka et al. (2016), Macierzyński et al. (2020)
9	Fragariin A	23.9	220		1018	1691, 1567, 1265, 1209, 935 (galloyl‐bis‐HHDP‐glucose), 783 (bis‐HHDP‐glucose), 633 (galloyl‐HHDP‐glucose), and **301** (ellagic acid)	C_89_H_58_O_57_	1018.0770	1018.0747	2.26	Karlińska et al. (2019)
10	Lambertianin C without one HHDP moiety	24.2	217		1250	2200 ([M–H–HHDP]^−^), 1869 ([M–H]^−^–galloyl‐HHDP‐glucose), 1567 ([M–H–castalagin)]^−^, 1235 (bis‐HHDP‐glucose‐galloyl‐ellagic acid), 633 (galloyl‐HHDP‐glucose), and **301** (ellagic acid)	C_109_H_74_O_70_	1250.1073	1250.1043	2.40	Sójka et al. (2016); Enomoto (2021)
11	Ellagic acid deoxyhexose	25.8	248	447		**301** (ellagic acid)	C_20_H_16_O_12_	447.0597	447.0569	6.29	Fernandes et al. (2011)
12	Agrimoniin	26.8	220		934	1103 (agrimonic acid), 1085 (1103–H_2_0), 936, 935 (galloyl‐bis‐HHDP‐glucose), 897, 783 (bis‐HHDP‐glucose), 633 (galloyl‐HHDP‐glucose), 613, and **301** (ellagic acid)	C_82_H_54_O_52_	934.0732	934.0718	1.50	Vrhovsek et al. (2012); Mena et al. (2012); Macierzyński et al. (2020)
13	Ellagic acid	27.8	250	301		–	C_14_H_6_O_8_	301.0007	300.9990	5.65	Gasperotti et al. (2013)
14	methyl ellagic acid hexoside^d^	28.8	249	477		346 and **301** (ellagic acid)	C_21_H_18_O_13_	477.0679	477.0675	0.84	Bubba et al. (2012)
15	ET unknown/galloyl‐castalagin isomer	30.5	210	1085		933 (castalagin), 783 (bis‐HHDP‐glucose), 633 (galloyl‐HHDP‐glucose), 451, and **301** (ellagic acid)		1085.0012			Mena et al. (2012); Gasperotti et al. (2013)
16	Dimethyl ellagic acid pentoside	32.3	245	461		446, 315, and **301** (ellagic acid)	C_21_H_18_O_12_	461.0734	461.0726	1.74	Bubba et al. (2012)

^a^ Ion used for fragmentation.

^b^ MS/MS ions marked in bold font represent fragments with an intensity more than 50% relative to the maximum intensity.

^c^ Monoisotopic mass; max wavelength [nm] detected from detector Knauer PDA 2800 used for quantification.

^d^ According to the literature data, it is not clear whether it is a methyl ellagic acid hexoside or a quercetin derivative.

**FIGURE 1:**
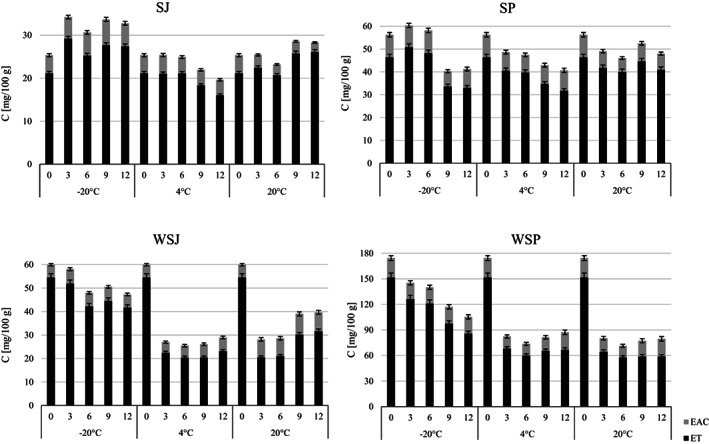
Stability of total ellagitannins (ET) and ellagic acid conjugates (EAC) during 12 months of storage of strawberry (SJ) and wild strawberry (WSJ) juices; and purees: Strawberry (SP) and wild strawberry (WSP)

Fresh juices produced from strawberries and wild strawberries contained 25 and 60 mg/100 g ETs + EAC, respectively (Figure 1). Purees of these fruits were characterized by more than twice and almost three times higher content of ETs + EAC.

The concentration of agrimoniin in the analyzed products varied. In fresh strawberry and wild strawberry juice, 4.7 and 3.5 mg/100 g of this compound were determined, respectively. Purees were characterized by higher agrimoniin content—in strawberry and wild strawberry, it was 12.5 and 34.3 mg/100 g, respectively. UEATr concentration was 31.6 and 57.6 mg/100 g in wild strawberry juice and wild strawberry puree, which accounted for 53 and 33% of the total sum of ETs and EAC. This compound was also present in strawberry juice and strawberry puree; however, it was present in much smaller amounts—2.8 and 3.8 mg/100 g, respectively. In strawberry juices and purees, the concentration of casuarictin, castalagin, and bis‐HHDP‐glucose constituted about 30% of the total ETs + EAC. In wild strawberry products, these compounds accounted for 20% of the total ETs + EAC.

The content of free ellagic acid was 2.5 and 2.7 mg/100 g in juices (strawberries and wild strawberries, respectively), and 6.7 and 14.2 mg/100 g in purees. The purees also showed a higher content of ellagic acid derivatives. The higher content of EA and its conjugates in purees may be related to the degradation of ellagitannins due to the rubbing process. Aaby et al. (2007), in a study on the effect of strawberry achenes on the content of polyphenols, note that an increase in achenes—from 0 to 2.9%—is accompanied by an increase in ellagic acid derivatives.

### 3.2 Stability of ETs during the storage of products

**FIGURE 2:**
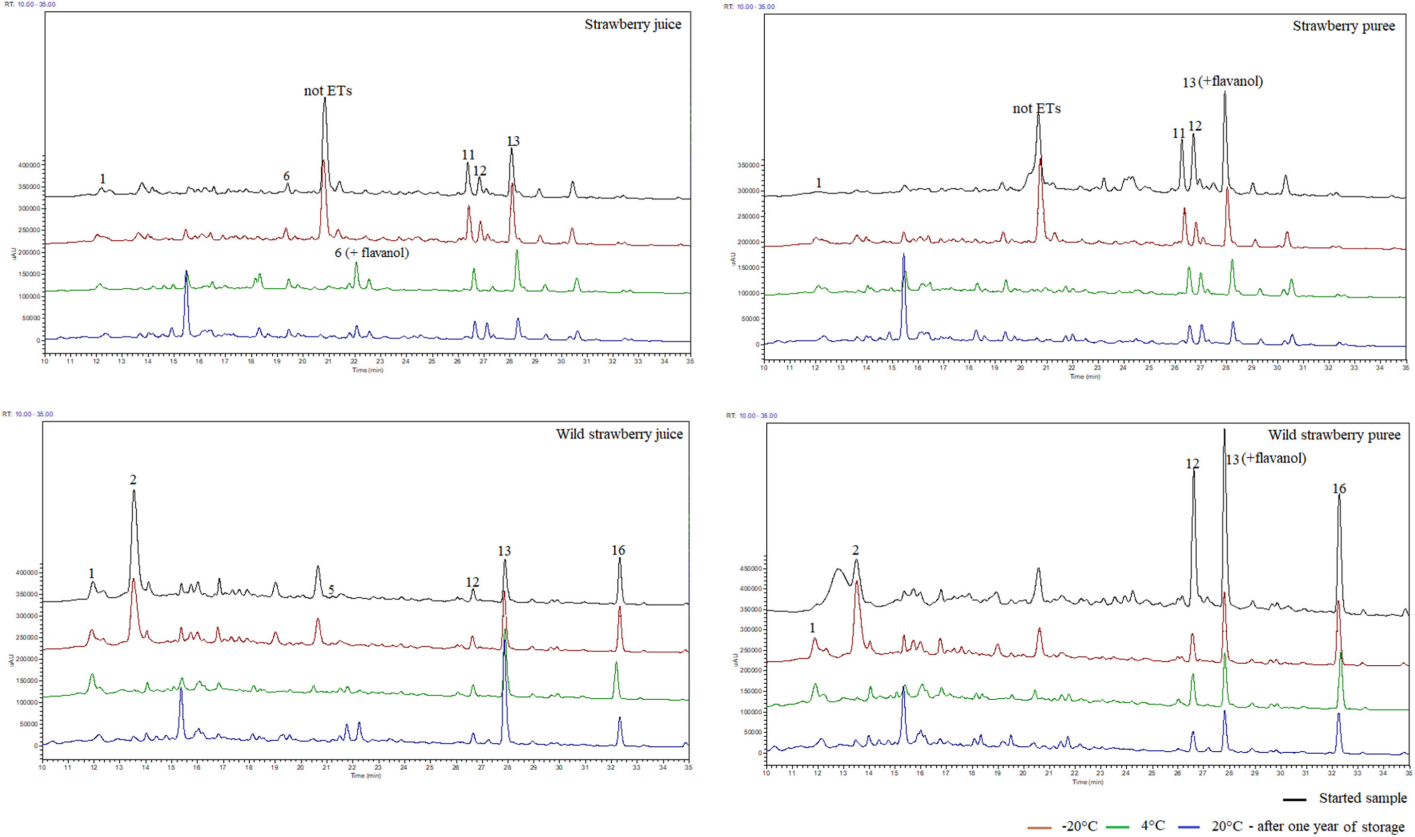
Chromatograms (250 nm) of juices and purees before and after 1 year of storage; marked major peaks correspond to Table 1

The results show that the stability of ETs + EAC is determined primarily by the temperature and storage time, as well as the matrix (Figure 2). After 1 year of frozen strawberry juice (SJ), an increase in the total concentration of ETs was noted—from 25 to 33 mg/100 g. The higher content of ETs and EA can be explained by the fact that, as a result of changes in the structure of the plant cell wall, these compounds are better released (Maas et al., 1991). The noticeable increase in the sum of the ETs + EAC at 20°C after 9 months in each case may be due to the extraction of these compounds from the solid particles. The decrease in ETs + EAC concentration was recorded at 4 and 20°C, by 30 and 20%, respectively, after 1 year of storage. Wild strawberry juice (WSJ) was less stable; after 12 months at −20, 4, and 20°C, the concentration of ETs decreased by 20, 50, and 30%, respectively. The rapid decrease in the sum of ETs and EAC conjugates concentration was mainly related to UEATr degradation or oxidation.

In the studies of Tiwari et al. (2009) on the stability of anthocyanins in strawberry juice, a rapid decrease in these compounds was noted during storage at 4 and 20°C. After 10 days of storage, 90.9 and 83.3% of the original anthocyanin content remained. The stability of ETs in the analyzed cases is much higher than that of anthocyanins.

In strawberry purees (SP), after 1 year of storage, the concentration of ETs + EAC was 41.2 and 40.7 mg/100 g at the temperature of −20 and 4°C and 48.0 mg/100 g at room temperature, which was 27, 28, and 15% lower, respectively, compared to the concentration of ETs + EAC in the fresh puree. After 12 months of storage in wild strawberry puree (WSP), 40, 49, and 54% less ETs + EAC were determined successively in relation to the concentration of these compounds in the fresh product for temperatures of −20, 4, and 20°C, respectively. The stability of strawberry puree was also investigated by other researchers. Aaby et al. (2007) noted a rapid decrease in the stability of the ETs during the storage of puree for 16 weeks at 22°C, after which more than 20% of the ETs + EAC had degraded. Researchers indicate the effect of achenes content on the stability of ETs + EAC. The concentration of ETs + EAC was stable when stored under the same puree conditions in the absence of achenes. In the puree without and with achenes, 80 and 72% of ETs + EAC were left, respectively. In the same study, a correlation was found between the concentration of other polyphenols and the rate of ellagitannin degradation.

The conducted analysis of Spearman's correlation (Table 2) showed that such a correlation between the time and the stability of the sum of ETs and EAC occurs in the case of strawberry and wild strawberry purees. The longer the purees were stored, the lower the ETs + EAC content was. For example, in strawberry puree, at 4 and 20°C, the concentration of ETs + EAC decreased on average by 6% every 3 months.

**TABLE 2:** Spearman's correlation—influence of time and temperature on the content of ellagitannins

	Temperature		Time		Temperature		Time	
	Juices	Purees	Juices	Purees	Strawberry	Wild strawberry	Strawberry	Wild strawberry
ETs^a^	−0.3007 *	−0.1509	−0.0609	−0.3074	−0.0767	−0.2464	−0.1219	−0.2372
EAC^b^	−0.1776	−0.1958	0.1104	−0.1726	−0.3050	0.0330	−0.1740	0.0794
Total ETs	−0.3113	−0.1462	−0.0432	−0.2900	−0.1250	−0.2169	−0.1586	−0.2192
LMW^c^	−0.1251	−0.0212	−0.0937	−0.2593	0.0348	−0.2222	−0.1277	−0.3111
HMW^d^	−0.2111	−0.4710	0.0334	−0.4323	−0.1934	−0.2258	−0.0892	−0.1491
UEATr^e^	−0.5785	−0.3192	−0.3800	−0.5484	−0.3884	−0.5120	−0.6290	−0.5040
Agrimoniin	−0.2033	−0.1864	0.0737	−0.2254	−0.2879	−0.2502	0.1254	−0.1939
Ellagic acid	−0.0827	−0.0430	0.0924	−0.1760	−0.2798	0.1239	−0.1421	0.1774

* The correlations were performed for *p* < 0.050—the results marked in red indicate a statistically significant correlation.

^a^ Ellagitannins.

^b^ Ellagic acid + ellagic acid conjugates.

^c^ Light molecular weight.

^d^ High molecular weight.

^e^ Unknown ellagic acid trimer derivative.

### 3.3 Stability of ETs depends on the molecular weight

The percentage ratio of oligomeric compounds to low‐molecular‐weight ETs can be a measure of the stability of the ETs during storage. Accordingly, all identified ETs were divided into high‐molecular‐weight (HMW) and low‐molecular‐weight (LMW) compounds. The HMW and LMW compounds for the wild strawberry fell within the weight ranges 1568–1870 Da and 302–1086 Da, respectively; for strawberries, the ranges of values were, respectively, 1266–2038 Da and 302–952 Da.

**FIGURE 3:**
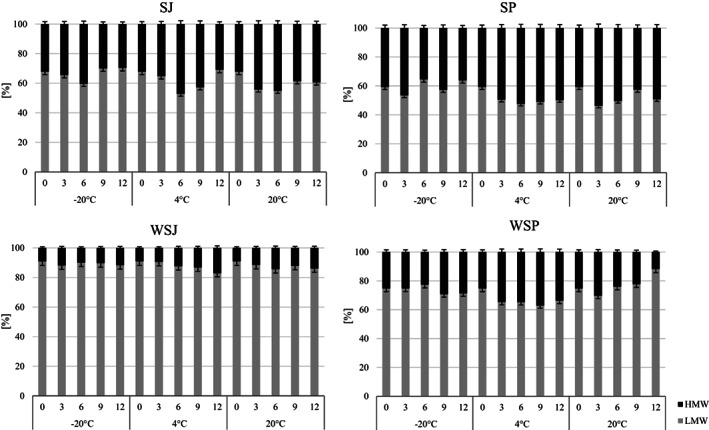
Ratio of high‐molecular‐weight (HMW) and low‐molecular‐weight (LMW) ETs during 12 months of storage of strawberry (SJ) and wild strawberry (WSJ) juices; and purees: strawberry (SP) and wild strawberry (WSP)

The LMW/HMW ratio in fresh strawberry products was 68/32 and 59/41 for juice and puree (Figure 3). In the strawberry juice and strawberry puree, the share of LMW/HMW compounds was 91/9 and 75/25, respectively. After 1 year of storage at −20°C, all the preserves had an LMW/HMW ratio similar to that of the fresh products. The increase in storage temperature resulted in varied size changes in the composition of ETs. At 4°C, the share of LMW compounds decreased on average by 10% for wild strawberry products and strawberry puree. At the same time, the share of HMW compounds increased. Changes in the LMW compound content are due to the decrease in UEATr, identified in the wild strawberry and strawberry. A similar situation was observed at the temperature of 20°C for strawberry products and wild strawberry juice. For wild strawberry puree, there was a 14% increase in LMW compounds after 1 year of storage at the same temperature.

**FIGURE 4:**
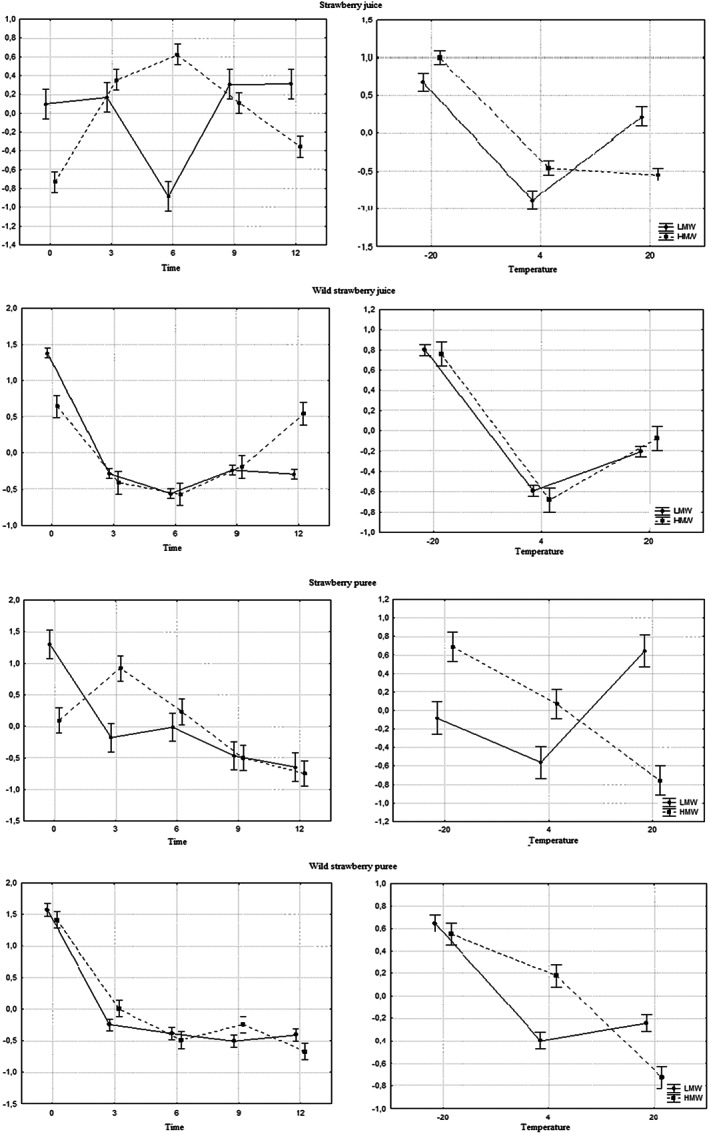
ANOVA factorial statistical analysis. **The analysis is based on standardized data. LMW, light‐molecular‐weight ellagitannins; HMW, high‐molecular‐weight ellagitannins

ANOVA statistical analysis (Figure 4) showed that the stability of high‐molecular‐weight ETs in wild strawberry products was influenced by the storage temperature the most. The analysis of the Spearman correlation (Table 2) for juices and purees showed that such a correlation exists between the temperature and storage time and the stability of high‐molecular‐weight ETs in purees (correlation −0.4710 and − 0.4323, respectively).

### 3.4 Transformations of ETs in juices and purees during storage

**FIGURE 5:**
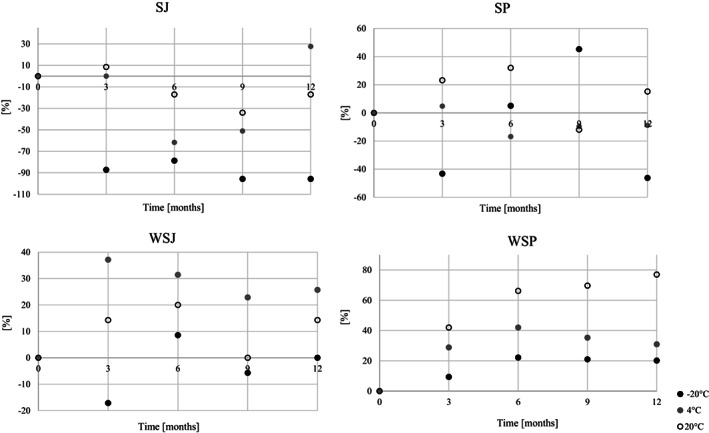
Percentage change in agrimoniin concentration during 12 months storage of strawberry (SJ) and wild strawberry (WSJ) juices; and purees: strawberry (SP) and wild strawberry (WSP)–positive values, decrease; and negative values, increase

The oligomeric compound identified in all products was agrimoniin. The content of this compound in juices and purees was varied. During the storage of the products, it was noticed that agrimoniin remained more stable in strawberry products as compared to wild strawberries (Figure 5). After 12 months of storage at the highest temperature, there was a 15% decrease for the puree and a 17% increase in this compound for the juice. The increase in the concentration of this ellagitannin may be caused by its extraction from solid particles present in the products. In the wild strawberry juice under the same conditions, agrimoniin concentration decreased by 15%. The decrease in this compound in the wild strawberry puree was much higher and amounted to 76%. It may be due to degradation or oxidation of the agrimoniin structure.

Previous studies on the stability of oligomeric ETs have been based on strong acid hydrolysis of pure isolated compounds (Macierzyński et al., 2020). The stability of high‐molecular‐weight ETs in purified extracts depending on pH and temperature was also tested (Sójka et al., 2019).

It has been shown that the main product of strong acid hydrolysis of agrimoniin is a compound with a molecular weight (MW) of 1568 Da, resulting from the detachment of one EA molecule (Macierzyński et al., 2020). It is most likely the sanguiin H‐10 (SH‐10) isomer. Macierzyński et al. (2020) noted that the share of this compound and its isomers during 1 hour of hydrolysis increased to 20% and then decreased. In our work, the content of this compound in the analyzed juices and purees was varied. In fresh strawberry and wild strawberry juice, the concentration was 0.6 and 0.7 mg/100 g, respectively. After 3 months of storing strawberry juice, the content of this compound was more than twice as high at −20°C. At the temperature of 20°C, after 6 and 12 months, there was a 30% increase in SH‐10 for strawberry juice. In the case of wild strawberry juice stored at −20 and 4°C, the concentration of SH‐10 was almost twice as high after 1 year of storage, while at the temperature of 20°C, there were no significant differences in the concentration of SH‐10. In the strawberry puree, the increase in this compound was noted at the temperature of −20°C after 3 months. At other temperatures, for different storage times, a downward trend in SH‐10 concentration was observed. In wild strawberry puree, the highest concentration of SH‐10 was recorded after 6 and 9 months of storage at 20°C, which may confirm the degradation or oxidation of agrimoniin.

In other cases, the decrease in SH‐10 concentration may indicate the degradation of this compound. Macierzyński et al. (2020) indicate that next, another EA molecule detaches from SH‐10, creating a secondary product—a compound with MW = 1266 Da. This ellagitannin was identified in the case of wild strawberry juice and puree. The concentration of ETs with an MW equal to 1266 Da was varied, but there was a trend that the higher the puree storage temperature, the higher the concentration of this compound. In the case of strawberries, the presence of this compound was not found in any product throughout the storage period.

Macierzyński et al. (2020) state that parallel to the formation of ETs with MW = 1266 Da, bis‐HHDP‐glucose is formed most likely as a result of hydrolysis of the glycosidic bond on the first glucose carbon in the 1568 Da molecule. Bis‐HHDP‐glucose was identified in each of the analyzed products. In strawberry juices, the concentration of this compound increased with the time and temperature of storage. In fresh juice, the content of bis‐HHDP‐glucose was at level 2.5 mg/100 g, and after 1 year of storage at −20, 4, and 20°C, the content was at level 5.4, 4.0, and 12.3 mg/100 g, respectively. In the puree, the concentration of this compound increased until the 9th month of storage, and after 12 months it decreased regardless of the storage temperature. In wild strawberry juice, the highest concentration of bis‐HHDP‐glucose was recorded for the highest temperature and the longest storage time—the concentration of this compound increased from 9.1 to 14 mg/100 g. In the case of wild strawberry puree, the highest concentration of bis‐HHDP‐glucose was recorded after 3 months for temperatures 20 and 4°C. At the temperature of 20°C, after 6 months there was a 20% increase in this compound, and over the course of 9 and 12 months, this concentration gradually decreased.

As a result of the hydrolysis of ETs with MW = 1266 Da, agrimonic acid with MW = 1104 Da may also be formed (Macierzyński et al., 2020). In the tested products, this relationship was not identified in any case. This is probably due to the fact that the preserves are not stored in such drastic conditions as acid hydrolysis.

The products of agrimoniin hydrolysis are also gal‐HHDP‐glucose and casuarictin with masses of 634 and 936 Da, respectively (Macierzyński et al., 2020). No gal‐HHDP‐glucose was found in juices and purees. The casuarictin content varied depending on the product, storage time, and temperature. The highest increase in the concentration of this compound was recorded in the strawberry juice after 3 months of storage at 4 and 20°C. In the strawberry puree, the casuarictin content decreased with the increase in time in each temperature variant. In wild strawberry products, the concentration of casuarictin varied throughout the storage period at each temperature.

As a result of the strong acid hydrolysis of agrimoniin, more than 20 secondary compounds can be formed under certain conditions. Moreover, it has been proven that secondary products for oligomeric ETs with different structures can be the same (Macierzyński et al., 2020). In the case of juices and purees analyzed in this study, in most cases (except for wild strawberry puree), agrimoniin was quite stable with a simultaneous increase in secondary products. This may indicate the decrease in other high‐molecular‐weight ellagitannins, e.g., the sanguiin H‐6 (SH‐6) isomer. In the considered products, SH‐6 was identified, in the amount of 1.6 and 1.2 mg/100 g, respectively, for strawberry and wild strawberry juices and 6.9 and 6.7 for purees.

**FIGURE 6:**
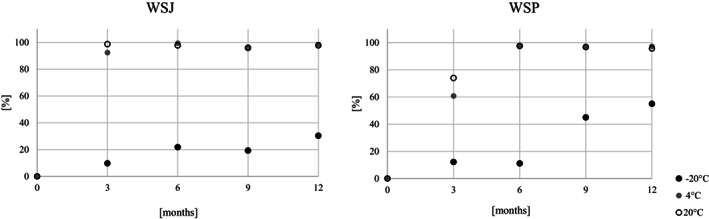
Percentage decrease in dominant compound (UEATr) during 12 months of storage of wild strawberry juice (WSJ) and puree (WSP)

Ellagic acid and its conjugates are formed by the degradation of high‐molecular‐weight ETs, in addition to intermediate products. In the case of strawberry preparations, a decrease in ellagic acid was noted during storage. Variable trends in the concentration of EA in the analyzed preserves may be related primarily to the decrease in UEATr, occurring mainly in the wild strawberry (Figure 6). This compound decreased very quickly, regardless of time and temperature. This is also indicated by the Spearman correlation—the stability of this compound was highly negatively correlated with time and temperature for both juices and purees.

In a study on the stability of strawberry purees stored for 16 weeks at 22°C, Aaby et al. (2007) noted a twofold increase in the concentration of EA. However, these researchers noticed that the concentration of ellagic acid may be underestimated due to the low solubility of this compound in water. When storing strawberries for 9 months at −20°C, a 40% decrease in total ellagic acid was observed, which can be explained by the ability of ETs to chelate metals and to react with free radicals (Häkkinen et al., 2000). In a study on raspberry jam, a twofold increase in ellagic acid was noted during 1‐month storage at 20°C and a decrease in this compound over the next 2 months (Zafrilla et al., 2001). In addition, as a result of damage to plant cells, enzymes are also released, including polyphenol oxidases, which may result in the loss of ellagic acid (De Ancos et al., 2000). In the case of the analyzed wild strawberry products, similar trends were observed; in the preparations stored for 12 months at the highest temperature, the EA content increased from 2.7 to 6.3 mg/100 g for wild strawberry juice and from 14.2 to 15.7 mg/100 g for wild strawberry puree.

## 4 CONCLUSIONS

The matrix may have an influence on the stability of ETs. The presence of acids, minerals, proteins, polysaccharides, pigments. and other polyphenolic compounds can modify the stability of these compounds. The innovation was to compare two different products with a variable composition in quantity and quality ETs coming from two different fruits (strawberry and wild strawberry).

Studies on the stability of ETs in unclarified juices and purees have shown that these compounds are susceptible to degradation/oxidation. The stability of ETs depends on the temperature and storage time as well as the matrix. With increasing temperature and storage time, the degree of decrease in ETs increased in each case studied. After 1 year of storing strawberry preserves, a 15–30% decrease in ETs was noted, depending on the temperature. However, there was no clear trend visible, which may indicate the relatively high stability of the ETs. 20–54% of the total ETs have decreased in wild strawberry products. At higher temperatures, a 10–14% increase in the proportion of compounds with a lower molecular weight was observed. At −20°C, the ratio of low‐ and high‐molecular‐weight compounds remained stable in each case under consideration.

The determination of changes in the ratio of low‐ and high‐molecular‐weight ETs and changes in ETs during storage allows the bioavailability of this group of polyphenols to be understood and their health‐promoting properties to be studied.

## FUNDING INFORMATION

This research did not receive any specific grant from funding agencies in the public, commercial, or not‐for‐profit sectors.

## CONFLICT OF INTEREST

The authors declare that they do not have any conflict of interest.

## ETHICAL APPROVAL

This study does not involve any human or animal testing.

## Supporting information


Table S1.

## Data Availability

The data that support the findings of this study are available on request from the corresponding author.

## References

[fsn33172-bib-0001] Aaby, K. , Wrolstad, R. E. , Ekeberg, D. , & Skrede, G. (2007). Polyphenol composition and antioxidant activity in strawberry purees; impact of achene level and storage. Journal of Agricultural and Food Chemistry, 55, 5156–5166. 10.1021/jf070467u 17550269

[fsn33172-bib-0002] Al‐Zoreky, N. S. (2009). Antimicrobial activity of pomegranate (*Punica granatum* L.) fruit peels. International Journal of Food Microbiology, 134, 244–248. 10.1016/j.ijfoodmicro.2009.07.002 19632734

[fsn33172-bib-0003] Ascacio‐Valdés, J. , Burboa, E. , Aguilera‐Carbo, A. F. , Aparicio, M. , Pérez‐Schmidt, R. , Rodríguez, R. , & Aguilar, C. N. (2013). Antifungal ellagitannin isolated from *Euphorbia antisyphilitica* Zucc. Asian Pacific Journal of Tropical Biomedicine, 3(1), 41–46. 10.1016/S2221-1691(13)60021-0 23570015 PMC3609384

[fsn33172-bib-0004] Beekwilder, J. , Jonker, H. , & Meesters, P. (2005). Antioxidants in raspberry: On‐line analysis links antioxidant activity to a diversity of individual metabolites. Journal of Agricultural and Food Chemistry, 53(9), 3313–3320. 10.1021/jf047880b 15853365

[fsn33172-bib-0005] Bubba, M. , Checchini, L. , Chiuminatto, U. , Doumett, S. , Fibbi, D. , & Giordani, E. (2012). Liquid chromatographic/electrospray ionization tandem mass spectrometric study of polyphenolic composition of four cultivars of *Fragaria vesca* L. berries and their comparative evaluation. Journal of Mass Spectrometry, 47, 1207–1220. 10.1002/jms.3030 22972789

[fsn33172-bib-0006] Clifford, M. N. , & Scalbert, A. (2000). Review: Ellagitannins‐nature, occurrence and dietary burden. Journal of the Science of Food and Agriculture, 80, 1118–1125.

[fsn33172-bib-0007] Corthout, J. , Peiters, L. A. , Claeys, M. , Vanden Berghe, D. A. , & Vlietinck, A. J. (1991). Antiviral ellagitannins from Spondias mombin. Phytochemistry, 30, 1129–1130. 10.1016/S0031-9422(00)95187-2

[fsn33172-bib-0008] De Ancos, B. , Gonzalez, E. M. , & Cano, M. P. (2000). Ellagic acid, vitamin C and total phenolic contents and radical scavenging capacity affected by freezing and frozen storage in raspberry fruit. Journal of Agricultural and Food Chemistry, 48, 4565–4570. 10.1021/jf0001684 11052701

[fsn33172-bib-0009] Enomoto, H. (2021). Unique distribution of ellagitannins in ripe strawberry fruit revealed by mass spectrometry imaging. Current Research in Food Science, 4, 821–828. 10.1016/j.crfs.2021.11.006 34841268 PMC8606305

[fsn33172-bib-0010] Fernandes, A. , Sousa, A. , Mateus, N. , Cabral, M. , & de Freitas, V. (2011). Analysis of phenolic compounds in cork from *Quercus suber* L. by HPLC–DAD/ESI–MS. Food Chemistry, 125, 1398–1405. 10.1016/j.foodchem.2010.10.016

[fsn33172-bib-0011] García‐Villalba, R. , Espín, J. C. , Aaby, K. , Alasalvar, C. , Heinonen, M. , Jacobs, G. , Voorspoels, S. , Koivumaki, T. , Kroon, P. A. , Pelvan, E. , Saha, S. , & Tomas‐Barberan, F. A. (2015). Validated method for the characterization and quantification of extractable and nonextractable ellagitannins after acid hydrolysis in pomegranate fruits, juices, and extracts. Journal of Agricultural and Food Chemistry, 63, 6555–6566. 10.1021/acs.jafc.5b02062 26158321

[fsn33172-bib-0012] Gasperotti, M. , Masuero, D. , Guella, G. , Palmieri, L. , Martinatti, P. , Pojer, E. , Mattivi, F. , & Vrhovsek, U. (2013). Evolution of ellagitannin content and profile during fruit ripening in *Fragaria* spp. Journal of Agricultural and Food Chemistry, 61, 8597–8607. 10.1021/jf402706h 23992396

[fsn33172-bib-0013] Gasperotti, M. , Masuero, D. , Vrhovsek, U. , Guell, G. , & Mattivi, F. (2010). Profiling and accurate quantification of *Rubus* ellagitannins and ellagic acid conjugates using direct UPLC‐Q‐TOF HDMS and HPLC‐DAD analysis. Journal of Agricultural and Food Chemistry, 58, 4602–4616. 10.1021/jf904543w 20353173

[fsn33172-bib-0014] Hager, T. J. , Howard, L. R. , Liyanage, R. , Lay, J. O. , & Prior, R. L. (2008). Ellagitannin composition of blackberry as determined by HPLC‐ESIMS and MALDI‐TOF‐MS. Journal of Agricultural and Food Chemistry, 56, 661–669. 10.1021/jf071990b 18211030

[fsn33172-bib-0015] Häkkinen, S. H. , Karenlampi, S. O. , Mykkanen, H. M. , Heinonen, I. M. , & Torronen, A. R. (2000). Ellagic acid content in berries: Influence of domestic processing and storage. European Food Research and Technology, 212, 75–80. 10.1007/s002170000184

[fsn33172-bib-0016] Heber, D. (2008). Multitargeted therapy of cancer by ellagitannins. Cancer Letters, 269, 262–268. 10.1016/j.canlet.2008.03.043 18468784

[fsn33172-bib-0017] Jurgoński, A. , Juśkiewicz, J. , Fotschki, B. , Kołodziejczyk, K. , Milala, J. , Kosmala, M. , Grzelak‐Błaszczyk, K. , & Markiewicz, L. (2017). Metabolism of strawberry mono‐ and dimeric ellagitannins in rats fed a diet containing fructo‐oligosaccharides. European Journal of Nutrition, 56, 853–864. 10.1007/s00394-015-1133-5 26689795 PMC5334382

[fsn33172-bib-0018] Kahkonen, M. , Kylli, P. , Ollilainen, V. , Salminen, J.‐P. , & Heinonen, M. (2012). Antioxidant activity of isolated ellagitannins from red raspberries and cloudberries. Journal of Agricultural and Food Chemistry, 60, 1167–1174. 10.1021/jf203431g 22229937

[fsn33172-bib-0019] Karlińska, E. , Pecio, Ł. , Macierzyński, J. , Stochmal, A. , & Kosmala, M. (2019). Structural elucidation of the ellagitannin with a molecular weight of 2038 isolated from strawberry fruit (*Fragaria ananassa* Duch.) and named fragariin a. Food Chemistry, 296, 109–115. 10.1016/j.foodchem.2019.05.191 31202294

[fsn33172-bib-0020] Klewicka, E. , Sójka, M. , Klewicki, R. , Kołodziejczyk, K. , Lipińska, L. , & Nowak, A. (2016). Ellagitannins from raspberry (*Rubus idaeus* L.) fruit as natural inhibitors of *Geotrichum candidum* . Molecules, 21, 908. 10.3390/molecules21070908 27420041 PMC6273995

[fsn33172-bib-0021] Klewicka, E. , Sójka, M. , Ścieszka, S. , Klewicki, R. , Milczarek, A. , Lipińska, L. , & Kołodziejczyk, K. (2020). The antimycotic efect of ellagitannins from raspberry (*Rubus idaeus* L.) on *Alternaria alternata* ŁOCK 0409. European Food Research and Technology, 246, 1341–1349. 10.1007/s00217-020-03493-0

[fsn33172-bib-0022] Klumpers, J. , Scalbert, A. , & Janin, G. (1994). Ellagitannins in european oak wood: Polymerization during wood aging. Phytochemistry, 36, 1249–1252. 10.1016/S0031-9422(00)89646-6

[fsn33172-bib-0023] Ko, H. , Hyelin, J. , Dahae, L. , Hyo‐Kyoung, C. , Ki, S. K. , & KyungChul, C. (2015). Sanguiin H6 suppresses TGF‐b induction of the epithelial− mesenchymal transition and inhibits migration and invasion in A549 lung cancer. Bioorganic & Medicinal Chemistry Letters, 25, 5508–5513. 10.1016/j.bmcl.2015.10.067 26508552

[fsn33172-bib-0024] Lansky, E. P. , Jiang, W. , Mo, H. , Bravo, L. , Froom, P. , Yu, W. , Harris, N. M. , Neeman, I. , & Campbell, M. J. (2005). Possible synergistic prostate cancer suppression by anatomically discrete pomegranate fractions. Investigational New Drugs, 23, 11–20. 10.1016/S0031-9422(00)89646-6 15528976

[fsn33172-bib-0025] Maas, J. L. , Wang, S. Y. , & Galletta, G. J. (1991). Evaluation of strawberry cultivars for ellagic acid content. HortScience, 26, 66–68. 10.21273/HORTSCI.26.1.66

[fsn33172-bib-0026] Macierzyński, J. , Sójka, M. , Kosmala, M. , & Karlińska, E. (2020). Transformation of oligomeric ellagitannins, typical for *Rubus* and *Fragaria* genus, during strong acid hydrolysis. Journal of Agricultural and Food Chemistry, 68(31), 8212–8222. 10.1021/acs.jafc.0c02674 32648752 PMC7458417

[fsn33172-bib-0027] Mena, P. , Calani, L. , Dall'Asta, C. , Galaverna, G. , García‐Viguera, C. , Bruni, R. , Crozier, A. , & Del Rio, D. (2012). Rapid and comprehensive evaluation of (poly) phenolic compounds in pomegranate (*Punica granatum* L.) juice by UHPLC‐MSn. Molecules, 12(148), 21–40. 10.3390/molecules171214821 PMC6268091 23519255

[fsn33172-bib-0028] Milczarek, A. , Sójka, M. , & Klewicki, R. (2021). Transfer of ellagitannins to unclarified juices and purees in the processing of selected fruits of the *Rosaceae* family. Food Chemistry, 344, 128684. 10.1016/j.foodchem.2020.128684 33272756

[fsn33172-bib-0029] Piekarska‐Radzik, L. , Klewicka, E. , Milala, J. , Klewicki, R. , Rosół, N. , Matysiak, B. , Sójka, M. , & Markowski, J. (2019). Wpływ polifenoli z wytłoków pseudoowoców *Rosa rugosa* Thunb. na wzrost bakterii z rodzaju *Lactobacillus* . ŻYWNOŚĆ Nauka Technologia Jakość, 26(3), 73–87. 10.15193/zntj/2019/120/298

[fsn33172-bib-0030] Sangiovanni, E. , Vrhovsek, U. , Rossoni, G. , Colombo, E. , Brunelli, C. , Brembati, L. , Trivulzio, S. , Gasperotti, M. , Mattivi, F. , Bosisio, E. , & Dell'Agli, M. (2013). Ellagitannins from Rubus berries for the control of gastric inflammation: In vitro and In vivo studies. PLoS One, 8(8), e71762. 10.1371/journal.pone.0071762 23940786 PMC3733869

[fsn33172-bib-0031] Sójka, M. , Janowski, M. , & Grzelak‐Błaszczyk, K. (2019). Stability and transformations of raspberry (*Rubus idaeus* L.) ellagitannins in aqueous solutions. European Food Research and Technology, 245, 1113–1122. 10.1007/s00217-018-3212-3

[fsn33172-bib-0032] Sójka, M. , Macierzyński, J. , Zaweracz, W. , & Buczek, M. (2016). Transfer and mass balance of ellagitannins, anthocyanins, flavan‐3‐ ols, and flavonols during the processing of red raspberries (*Rubus idaeus* L.) to juice. Journal of Agricultural and Food Chemistry, 64, 5549–5563. 10.1021/acs.jafc.6b01590 27292440

[fsn33172-bib-0033] Tiwari, B. K. , O'Donnell, C. P. , Patras, A. , et al. (2009). Stability of anthocyanins and ascorbic acid in sonicated strawberry juice during storage. European Food Research and Technology, 228, 717–724. 10.1007/s00217-008-0982-z

[fsn33172-bib-0034] Viriot, C. , Sclabert, A. , Herve du Penhoat, C. L. M. , & Moutounet, M. (1994). Ellagitannins in wood of sessile oak and sweet chestnut dimerization and hydrolysis during wood aging. Phytochemistry, 36, 1253–1260. 10.1016/S0031-9422(00)89647-8

[fsn33172-bib-0035] Viriot, C. , Sclabert, A. , Lapierre, C. , & Moutounet, M. (1993). Ellagitannins and lignins in aging of spirits in oak barrels. Journal of Agricultural and Food Chemistry, 41, 1872–1879. 10.1021/jf00035a013

[fsn33172-bib-0036] Vrhovsek, U. , Guella, G. , Gasperotti, M. , Pojer, E. , Zancato, M. , & Mattivi, F. (2012). Clarifying the identity of the main ellagitannin in the fruit of the strawberry, *Fragaria vesca* and *Fragaria ananassa* Duch. Journal of Agricultural and Food Chemistry, 60, 2507–2516. 10.1021/jf2052256 22339338

[fsn33172-bib-0037] Zafrilla, J. P. , Ferreres, F. , & Tomas‐Barberan, F. A. (2001). Effect of processing and storage on the antioxidant ellagic acid derivatives and flavonoids of red raspberry (*Rubus idaeus*). Journal of Agricultural and Food Chemistry, 49, 3651–3655. 10.1021/jf010192x 11513642

