# Supplementary material for: Stability of ellagitannins in processing products of selected Fragaria fruit during 12 months of storage
Source: Food Sci Nutr. 2022 Dec 22;11(3):1354–66. doi: 10.1002/fsn3.3172 (PMC10003011; doi:10.1002/fsn3.3172)
Supplement: Supplementary file 1 — Table S1. [file FSN3-11-1354-s001.docx]

Supplementary material

S1. Elagitannins in unclarified juices and purees

| **Comp.** | **1** | **2** | **3** | **4** | **5** | **6** | **7** | **8** | **9** | **10** | **11** | **12** | **13** | **14** | **15** | **16** | **TOTAL** |
| --- | --- | --- | --- | --- | --- | --- | --- | --- | --- | --- | --- | --- | --- | --- | --- | --- | --- |
| months | **starting SJ^a.^** | | | | | | | | | | | | | | | | |
| 0 | 2.5±0.01a | 2.8±0.10f | 2.6±0.04e | nd | 0.6±0.10bc | 2.5±0.12ef | 2.8±0.01e | 1.6±0.02f | 0.3±0.02ab | 0.7±0.02c | 1.7±0.01e | 4.7±0.02b | 2.5±0.01ef | nd | nd | nd | 25.4±0.14c |
| **SJ -20°C** | | | | | | | | | | | | | | | | | |
| 3 | 4.5±0.08d | 3.3±0.13h | 3.6±0.05g | nd | 1.4±0.22e | 2.6±0.06f | 0.7±0.09b | 1.3±0.00d | 2.2±0.00h | 0.7±0.14c | 2.0±0.00f | 8.8±0.06g | 3.0±0.05g | nd | nd | nd | 34.2±0.05f |
| 6 | 3.1±0.11b | 2.7±0.12e | 3.9±0.12h | nd | 1.0±0.26d | 1.7±0.02b | 0.5±0.09ab | 1.6±0.09ef | 1.7±0.10g | 0.7±0.06c | 2.0±0.03f | 8.4±0.08g | 3.0±0.02g | 0.4±0.02b | nd | nd | 30.7±0.17e |
| 9 | 4.7±0.07de | 3.6±0.11i | 3.6±0.03g | nd | 1.0±0.04d | 1.5±0.03b | 1.4±0.04d | 1.1±0.06c | 0.5±0.04c | 1.2±0.04e | 2.2±0.05g | 9.2±0.21h | 3.3±0.07h | 0.5±0.02b | nd | nd | 33.7±0.81f |
| 12 | 5.4±0.17f | 3.1±0.02g | 3.2±0.28f | nd | 1.0±0.01d | 1.4±0.05b | 1.4±0.00d | 1.1±0.01c | 0.4±0.01bc | 1.2±0.02e | 2.0±0.03f | 9.2±0.15h | 3.4±0.07h | nd | nd | nd | 32.8±0.78f |
| **SJ 4°C** | | | | | | | | | | | | | | | | | |
| 3 | 5.7±0.23g | 0.8±0.05d | 0.8±0.09a | nd | 0.3±0.05ab | 1.9±0.20c | 3.8±0.23g | 1.7±0.01f | 1.3±0.08f | nd | 1.7±0.18e | 4.7±0.59b | 2.7±0.21f | nd | nd | nd | 25.4±1.74c |
| 6 | 4.9±0.09ef | 0.3±0.03b | 2.0±0.04cd | nd | 0.5±0.03bc | 2.1±0.22cd | 0.4±0.08a | 1.4±0.08e | 1.3±0.01f | 0.4±0.05b | 1.5±0.03d | 7.6±0.12f | 1.9±0.02d | 0.4±0.01b | nd | nd | 24.9±0.20c |
| 9 | 5.4±0.08fg | nd | 1.0±0.02a | nd | 0.4±0.02ab | 1.6±0.08b | 1.0±0.02c | 0.5±0.03a | 0.2±0.01a | 1.1±0.02e | 1.4±0.12c | 7.1±0.09e | 1.8±0.02d | 0.4±0.01b | nd | nd | 21.9±0.38b |
| 12 | 4.0±0.22c | 0.4±0.02c | 0.9±0.02a | nd | 0.2±0.01a | 4.3±0.36g | 0.4±0.01a | 1.1±0.11c | 0.4±0.07abc | 0.9±0.06d | 1.2±0.06b | 3.4±0.11a | 2.4±0.14e | nd | nd | nd | 19.6±1.19a |
| **SJ 20°C** | | | | | | | | | | | | | | | | | |
| 3 | 7.3±0.11h | 0.2±0.02ab | 1.5±0.09b | nd | 0.6±0.01bc | 2.4±0.01def | 3.1±0.03f | 1.6±0.01f | 1.4±0.18f | nd | 1.4±0.02c | 4.3±0.09b | 1.5±0.00c | nd | nd | nd | 25.4±0.26c |
| 6 | 9.3±0.17i | 0.1±0.03a | 1.3±0.14b | nd | 0.9±0.03d | 0.6±0.07a | 0.4±0.04a | 1.2±0.14cd | 1.1±0.14e | 0.3±0.03a | 1.0±0.02a | 5.5±0.28c | 1.3±0.05ab | 0.3±0.02a | nd | nd | 23.2±1.14b |
| 9 | 11.3±0.65j | 0.3±0.03b | 2.3±0.23d | nd | 0.4±0.03ab | 2.3±0.04de | 1.0±0.03c | 0.5±0.02a | 0.3±0.02ab | 1.1±0.06e | 1.1±0.04b | 6.3±0.10d | 1.4±0.02bc | 0.3±0.03a | nd | nd | 28.6±1.26d |
| 12 | 12.3±0.17k | nd | 1.9±0.06c | nd | 0.8±0.01cd | 2.2±0.04cd | 1.4±0.06ds | 0.7±0.01b | 0.8±0.08d | 0.6±0.02c | 1.0±0.03a | 5.5±0.20c | 1.2±0.02a | nd | nd | nd | 28.3±0.67d |
| **starting SP^b^** | | | | | | | | | | | | | | | | | |
| 0 | 4.4±0.65a | 3.8±0.09e | 4.2±0.36g | nd | 1.1±0.16bc | 6.5±0.10g | 5.0±0.43c | 6.9±0.05g | 0.6±0.04a | 1.3±0.05def | 3.2±0.06f | 12.5±0.02cd | 6.7±0.14e | nd | nd | nd | 56.3±1.94e |
| **SP -20°C** | | | | | | | | | | | | | | | | | |
| 3 | 6.3±0.02b | 3.9±0.05e | 4.4±0.06g | nd | 1.6±0.05e | 3.7±0.30bcd | 0.8±0.05a | 3.2±0.01ef | 7.3±0.09f | 1.7±0.05fg | 3.1±0.08f | 17.9±0.04f | 6.4±0.12e | nd | nd | nd | 60.3±0.35e |
| 6 | 8.3±0.03d | 3.1±0.09d | 4.3±0.19g | nd | 1.1±0.02bcd | 3.2±0.14a | 0.8±0.16a | 3.4±0.02f | 5.6±0.21de | 1.4±0.05de | 2.8±0.02e | 17.0±0.23f | 6.4±0.16e | 0.8±0.12e | nd | nd | 58.1±0.13e |
| 9 | 6.3±0.43bc | nd | 3.4±0.06ef | nd | 0.7±0.14a | 3.4±0.19ab | 0.6±0.12a | 3.0±0.14cde | 2.6±0.21c | 0.9±0.07cde | 2.4±0.07d | 14.0±0.14e | 4.8±0.07bc | 0.4±0.14b | nd | nd | 40.0±1.91abcd |
| 12 | 4.9±0.41a | nd | 3.1±0.03e | nd | 0.6±0.03a | 4.1±0.20de | 0.6±0.12bc | 2.5±0.13a | 2.1±0.04b | 1.5±0.13defg | 1.9±0.10b | 13.6±0.83de | 5.8±0.20d | 0.6±0.04cd | nd | nd | 41.2±2.20ab |
| **SP 4°C** | | | | | | | | | | | | | | | | | |
| 3 | 7.6±0.22cd | 0.6±0.01c | 1.0±0.05a | nd | 0.7±0.09a | 3.3±0.03ab | 4.2±0.30b | 2.7±0.20abc | 6.7±0.14f | 1.8±0.04g | 2.4±0.19d | 11.9±0.04c | 5.7±0.03d | nd | nd | nd | 48.7±0.09d |
| 6 | 8.6±0.06d | 0.3±0.04a | 2.3±0.09cd | nd | 0.6±0.02a | 3.6±0.11abc | 0.7±0.10ab | 2.9±0.10abcde | 5.3±0.02d | 0.8±0.08a | 2.2±0.06cd | 14.6±0.06e | 5.0±0.06b | 0.6±0.02cd | nd | nd | 47.5±1.10cd |
| 9 | 6.1±0.21b | nd | 1.9±0.14bc | nd | 0.7±0.07a | 4.2±0.21de | 0.6±0.15ab | 2.7±0.07abc | 3.8±0.14c | 1.2±0.07bcd | 2.0±0.21bc | 13.7±0.21de | 5.7±0.07cd | 0.7±0.06de | nd | nd | 43.0±0.92abc |
| 12 | 5.0±0.23a | nd | 1.3±0.10a | nd | 0.6±0.04a | 4.5±0.23e | 0.6±0.02ab | 2.5±0.12ab | 2.1±0.03b | 1.5±0.12efg | 1.9±0.09b | 13.6±0.82de | 6.6±0.34e | 0.6±0.04cd | nd | nd | 40.7±2.1a |
| **SP 20°C** | | | | | | | | | | | | | | | | | |
| 3 | 10.1±1.37e | 0.4±0.07ab | 1.8±0.16b | nd | 0.9±0.07b | 4.0±0.52cde | 4.0±0.41b | 2.8±0.43abcd | 6.1±0.84e | 2.2±0.30h | 2.0±0.30bc | 9.6±0.55ab | 5.3±0.59bcd | nd | nd | nd | 49.0±6.62d |
| 6 | 15.1±0.05g | nd | 3.6±0.24f | nd | 0.7±0.02a | 5.0±0.04f | nd | 3.1±0.07def | 2.5±0.26b | 0.9±0.20a | 1.6±0.04a | 8.5±0.08a | 4.0±0.03a | 0.5±0.03bc | nd | nd | 46.0±0.26bcd |
| 9 | 14.2±0.28fg | nd | 2.5±0.07d | nd | 1.2±0.07cd | 7.2±0.14h | 0.8±0.07ab | 2.9±0.14cde | 3.4±0.14b | 1.3±0.07ab | 1.4±0.14a | 9.3±0.28ab | 5.1±0.14b | 0.4±0.07bc | nd | nd | 52.4±1.70cd |
| 12 | 13.6±0.63f | nd | 1.3±0.03a | nd | 0.7±0.03a | 7.9±0.21i | 0.7±0.13a | 2.9±0.03cde | 2.4±0.19b | 0.8±0.23a | 1.4±0.03a | 10.6±0.31b | 5.3±0.06bcd | 0.4±0.02bc | nd | nd | 48.0±1.73cd |
| **starting WSJ^c^** | | | | | | | | | | | | | | | | | |
| 0 | 9.1±0.28bc | 31.6±0.09g | 2.3±0.44ef | 0.6±0.01b | 0.7±0.05c | 3.5±0.06de | 1.2±0.03cde | 1.2±0.03fg | nd | nd | nd | 3.5±0.04fg | 2.7±0.03ab | 0.3±0.03b | 0.7±0.03e | 2.5±0.03e | 60.0±1.09g |
| **WSJ -20°C** | | | | | | | | | | | | | | | | | |
| 3 | 7.4±0.76a | 28.5±1.04f | 1.6±0.10bcd | 0.8±0.01c | 0.7±0.05c | 3.3±0.31de | 2.5±0.37g | 1.5±0.12h | nd | nd | nd | 4.1±0.43h | 3.1±0.19bc | 0.4±0.14c | 0.8±0.09e | 2.7±0.08ef | 58.0±3.71g |
| 6 | 8.6±0.65b | 24.7±0.28e | 1.8±0.34cde | 0.3±0.02a | 0.4±0.03a | 1.5±0.06a | 0.2±0.03a | 1.2±0.04g | nd | nd | nd | 3.2±0.04ef | 2.9±0.06bc | 0.2±0.01a | 0.3±0.02c | 2.6±0.02ef | 48.0±1.53e |
| 9 | 6.8±0.10a | 25.5±0.43e | 2.1±0.04de | 0.8±0.01c | 0.6±0.02b | 2.6±0.07bc | 1.6±0.03f | 1.0±0.03de | nd | nd | nd | 3.7±0.06g | 3.2±0.06cd | 0.2±0.01a | nd | 2.7±0.04f | 50.5±0.83f |
| 12 | 7.2±0.17a | 22.0±0.86d | 2.2±0.09ef | 0.3±0.02a | 1.1±0.00e | 2.7±0.14c | 1.2±0.06c | 0.9±0.00d | nd | nd | nd | 3.5±0.05g | 3.0±0.00bc | nd | 0.6±0.00d | 2.6±0.11ef | 47.3±1.43e |
| **WSJ 4°C** | | | | | | | | | | | | | | | | | |
| 3 | 13.5±0.81g | 2.4±0.35c | 1.3±0.10abc | 0.3±0.03a | nd | 1.2±0.11a | 1.0±0.13c | 0.3±0.03ab | nd | nd | nd | 2.2±0.12a | 2.4±0.10a | nd | nd | 2.2±0.08cd | 27.0±1.86abc |
| 6 | 12.5±0.28f | 0.2±0.02a | 0.9±0.05a | 0.7±0.02c | 0.5±0.03b | 1.4±0.06a | 1.2±0.04cd | 0.3±0.01a | nd | nd | nd | 2.4±0.09ab | 2.9±0.05bc | nd | 0.2±0.04b | 2.2±0.03d | 25.5±0.69a |
| 9 | 11.0±0.58e | 1.1±0.01ab | 1.5±0.15bc | nd | 0.3±0.00a | 2.2±0.24b | 1.2±0.26cde | 0.4±0.03b | nd | nd | nd | 2.7±0.03cd | 3.6±0.04de | nd | nd | 2.1±0.03bc | 26.1±1.32ab |
| 12 | 10.5±0.12de | 0.5±0.01ab | 1.1±0.11ab | 1.3±0.09e | 1.3±0.11f | 3.3±0.47de | 1.0±0.02c | 1.1±0.09ef | nd | nd | nd | 2.6±0.06bc | 3.7±0.06e | nd | 0.3±0.00c | 2.2±0.02bcd | 29.0±1.07c |
| **WSJ 20°C** | | | | | | | | | | | | | | | | | |
| 3 | 9.2±0.57bc | 0.4±0.02a | 2.2±0.29ef | 1.0±0.11d | nd | 3.0±0.41cd | 1.4±0.11ef | 0.3±0.01a | nd | nd | nd | 3.0±0.15de | 5.6±0.31f | nd | nd | 2.0±0.11b | 28.1±2.09abc |
| 6 | 8.4±0.83b | 0.7±0.011ab | 3.1±0.40g | 0.2±0.02a | 0.9±0.06d | 3.3±0.39de | 1.2±0.11cde | 0.4±0.07ab | nd | nd | nd | 2.8±0.24cd | 5.7±0.43f | nd | 0.1±0.01a | 1.8±0.17a | 28.6±2.66bc |
| 9 | 9.8±0.09cd | 1.3±0.03b | 2.7±0.26fg | 1.0±0.03d | 0.5±0.04b | 9.1±0.36g | 1.4±0.05def | 0.8±0.05c | nd | nd | nd | 3.5±0.03fg | 7.1±0.6h | nd | nd | 1.8±0.03a | 39.0±0.93d |
| 12 | 14.0±0.25g | 0.7±0.01ab | 4.6±0.06g | 0.8±0.03c | 0.8±0.03c | 4.5±0.04f | 0.6±0.02b | 1.8±0.08i | nd | nd | nd | 3.0±0.05de | 6.3±0.04g | nd | 0.9±0.10f | 1.7±0.03a | 39.6±0.69d |
| **starting WSP^d^** | | | | | | | | | | | | | | | | | |
| 0 | 15.4±0.43abcd | 57.6±0.57f | 9.8±0.52e | 1.8±0.21cd | 3.0±0.09abc | 13.8±0.80f | 5.1±0.40g | 6.7±0.48f | nd | nd | nd | 34.3±1.04h | 14.2±0.13f | 0.8±0.03e | 4.0±0.13g | 7.7±0.03g | 174.3±4.81g |
| **WSP -20°C** | | | | | | | | | | | | | | | | | |
| 3 | 20.2±0.21ef | 50.6±0.35e | 5.6±0.26cd | 2.1±0.06de | 2.6±0.02bc | 6.0±0.78b | 2.8±0.09de | 2.9±0.39bc | nd | nd | nd | 31.1±0.65g | 11.8±0.00cd | 0.2±0.02a | 2.3±0.03f | 7.1±0.01efg | 145.3±2.73f |
| 6 | 17.1±0.03cd | 51.2±0.41e | 5.9±0.47d | 6.0±0.28i | 1.4±0.07abc | 6.1±0.52b | 1.9±0.32bc | 3.5±0.08cd | nd | nd | nd | 26.7±0.26ef | 11.0±0.13bc | 0.8±0.00e | 1.2±0.06e | 7.2±0.11fg | 140.1±2.78f |
| 9 | 15.15±0.87abcd | 31.7±2.12d | 4.8±0.25c | 3.7±0.21h | 0.7±0.14abc | 8.2±0.32c | 2.5±0.19cd | 2.7±0.14bc | nd | nd | nd | 27.1±0.14ef | 12.5±0.21de | 0.3±0.07b | 1.2±0.22e | 6.9±0.14ef | 117.1±1.83e |
| 12 | 13.0±3.06ab | 25.9±1.76c | 3.4±0.15b | 1.1±0.05b | 0.4±0.06ab | 8.8±0.52c | 2.8±0.21de | 2.3±0.08b | nd | nd | nd | 27.4±1.07f | 13.0±0.43e | nd | 0.6±0.05bcd | 6.6±0.27def | 105.4±7.80d |
| **WSP 4°C** | | | | | | | | | | | | | | | | | |
| 3 | 22.6±2.83f | 3.8±0.13b | 3.8±0.38b | 0.7±0.13b | 1.7±0.32abc | 4.4±0.37a | 3.2±0.36def | 2.4±0.35b | nd | nd | nd | 24.4±2.96de | 8.1±0.60a | nd | 1.0±0.16e | 6.3±0.77cde | 82.4±9.08bc |
| 6 | 16.6±0.38cd | 1.6±0.02a | 1.8±0.04a | 3.0±0.28g | 1.9±0.03abc | 9.0±0.26c | 1.6±0.00b | 3.8±0.03de | nd | nd | nd | 19.9±0.25c | 7.5±0.06a | 0.4±0.02d | 0.7±0.06cd | 5.9±0.15bcd | 73.6±1.58ab |
| 9 | 15.7±0.98abcd | 1.6±0.16a | 2.6±0.31a | 2.4±0.23ef | 1.6±0.13abc | 12.4±0.56e | 2.5±0.18cd | 4.1±0.09de | nd | nd | nd | 22.2±0.36cd | 10.1±0.12b | nd | 0.6±0.06abcd | 5.9±0.21bcd | 81.4±2.36bc |
| 12 | 14.6±2.92abc | 1.6±0.30a | 2.5±0.23a | 0.5±0.04a | 1.3±0.12abc | 14.1±1.40f | 3.7±0.57f | 4.4±0.40e | nd | nd | nd | 23.7±2.36d | 14.6±0.83f | nd | 0.4±0.03ab | 5.8±0.59bc | 87.2±9.76c |
| **WSP 20°C** | | | | | | | | | | | | | | | | | |
| 3 | 15.0±2.38abc | 1.7±0.33a | 9.3±1.14e | 2.5±0.36f | nd | 7.6±0.74c | 3.5±0.46ef | 4.4±0.66e | nd | nd | nd | 19.9±2.01c | 10.3±1.18b | nd | 0.4±0.09abc | 5.6±0.86abc | 80.3±9.44bc |
| 6 | 18.4±0.59de | 1.4±0.06a | 6.0±0.33d | 3.0±0.14g | 2.7±0.04c | 11.0±0.14d | 0.6±0.10a | 2.8±0.07bc | nd | nd | nd | 11.6±0.14b | 7.9±0.07a | 0.3±0.02c | 0.4±0.00cd | nd | 66.0±0.92a |
| 9 | 16.0±0.54bcd | 1.9±0.09a | 5.9±0.46d | 2.2±0.09def | 2.5±0.31bc | 15.8±0.39g | 1.4±0.12b | 2.4±0.12b | nd | nd | nd | 10.4±0.08ab | 13.4±0.21eg | nd | 0.8±0.09d | 5.3±0.23ab | 77.5±0.36bc |
| 12 | 12.4±0.02a | 2.5±0.05ab | 5.4±0.14cd | 1.5±0.03c | 0.7±0.00abc | 25.1±0.40h | 1.6±0.02b | 0.7±0.01a | nd | nd | nd | 7.9±0.07a | 15.7±0.84h | nd | 1.1±0.03e | 5.0±0.05a | 79.6±1.67bc |

Results are expressed in mg/100 g. ^a^ – strawberry juice, ^b^ – strawberry puree, ^c^ – wild strawberry juice, ^d^ – wild strawberry puree; no significant statistical differences were marked in individual columns within the product of a given fruit (temp * time) with the same lowercase letter (p> 0.05); peak numbers 1-14 correspond to the peaks in tab. 1; values ± standard deviation, n = 3
